# Water adsorption and dynamics on graphene and other 2D materials: Computational and experimental advances

**DOI:** 10.1080/23746149.2022.2134051

**Published:** 2022-11-11

**Authors:** M. Sacchi, A. Tamtögl

**Affiliations:** a Department of Chemistry, University of Surrey, Guildford GU2 7XH, UK; b Institute of Experimental Physics, Graz University of Technology, 8010 Graz, Austria

**Keywords:** Water, Ice, Water dynamics, Surface diffusion, Wettability, Graphene, 2D Materials, Topological insulators, Density functional theory, Surface scattering, Gas-surface dynamics, Energy dissipation

## Abstract

The interaction of water and surfaces, at molecular level, is of critical importance for understanding processes such as corrosion, friction, catalysis and mass transport. The significant literature on interactions with single crystal metal surfaces should not obscure unknowns in the unique behaviour of ice and the complex relationships between adsorption, diffusion and long-range inter-molecular interactions. Even less is known about the atomic-scale behaviour of water on novel, non-metallic interfaces, in particular on graphene and other 2D materials. In this manuscript, we review recent progress in the characterisation of water adsorption on 2D materials, with a focus on the nano-material graphene and graphitic nanostructures; materials which are of paramount importance for separation technologies, electrochemistry and catalysis, to name a few. The adsorption of water on graphene has also become one of the benchmark systems for modern computational methods, in particular dispersion-corrected density functional theory (DFT). We then review recent experimental and theoretical advances in studying the single-molecular motion of water at surfaces, with a special emphasis on scattering approaches as they allow an unparalleled window of observation to water surface motion, including diffusion, vibration and self-assembly.

**Figure:**
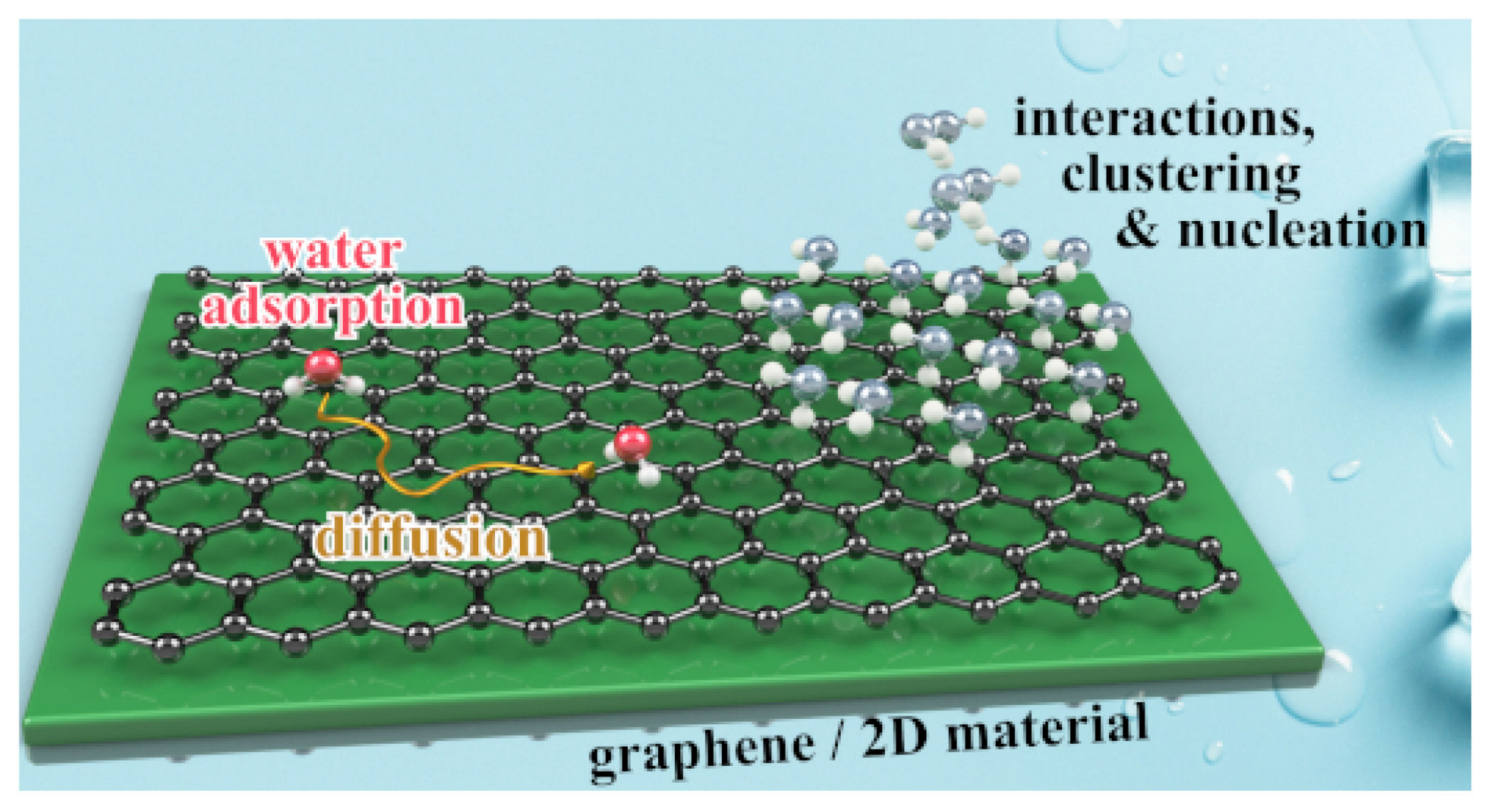

“If there is magic on this planet, it is contained in water.”

Loren Eiseley about Water^1^

## 1 Introduction

**Figure 1:**
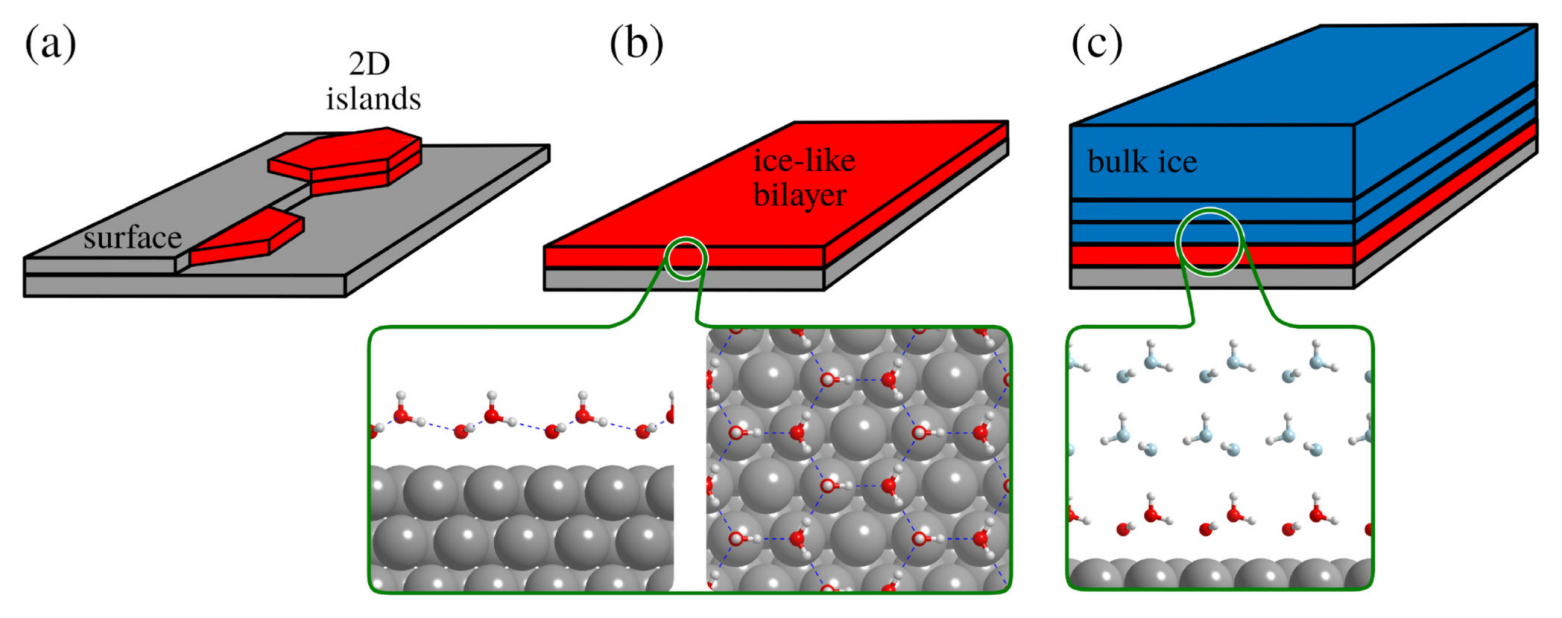
In order to understand the growth of water at surfaces and to develop models which cover the sub-monolayer (a) to thick-film regime (c), a molecular level understanding of the inital stages of water adsorption and the balance with inter-molecular forces is necessary. On flat metals surfaces, growth starts in 2D islands, with adsorption being strongly affected by nucleation sites in the form of steps and defects (a). The growth typically continues with a so-called bilayer-model[6] in (b) up to thick films in (c).

A vast amount of publications on the water molecule exists and makes H_2_O one of the most well known chemicals. Its extraordinary properties and in particular its capacity to form very strong intermolecular bonding through hydrogen bonds, makes it one of the most interesting and challenging molecular precursors for studying self-assembly, nucleation and growth of 2D and 3D crystals on surfaces. While hexagonal ice (ice I_h_) is the form of natural snow and ice on earth[1], H_2_O molecules at the interface with a surface, as illustrated in Figure 1, tend to adapt the structure of the underling substrate, giving rise to a multitude of structures due to an intricate interplay of molecule-surface and intermolecular interactions. Thus, water can form a variety of 2D ice structures on metallic and non-metallic surfaces[1–3], including so-called “hexagonal” or “pentagonal Ice” on Cu(110)[4,5], the famous $\sqrt{3}\times \sqrt{3}-R30^{{}^{\circ}}$ bilayer on Ru(0001) and *c*(2 × 2) over fcc metal surfaces (Ni, Pt, Ag, Cu)[6–10].

Thanks to the technological relevance of ice formation at surfaces to fields as diverse as aviation, wind power and telecommunications[11–13], the kinetics of nucleation and the growth of ice on surfaces are relatively well understood on the macroscopic scale - in stark contrast to the microscopic details of water dynamics and ice formation. Following the lines of Bartels-Rausch - “The chemistry and physics of ice need to be studied more on a molecular scale if we are to address the massive environmental problems we face.”[14] - we concentrate on two aspects in our review article: (i) The microscopic details of water adsorption on graphene and further emerging 2D materials and (ii) the diffusion of water on surfaces.

In the midst of a flourishing interest in wetting and anti-icing properties e.g. for designing superhydrophobic and anti-icing materials, diffusion of water on surfaces is a poorly understood behaviour: The atomistic details of the mechanism that governs surface diffusion of water remains largely unexplored, despite being a critical factor in a complex and multi-scale process that leads from adsorbed water monomers to ice. In this review we focus on recent progress in the understanding this process and the interaction of water with non-metal, non-bulk materials, in particular graphene and nanocarbons.

**Water adsorption and structure on graphene and novel surfaces:** One reason for the demand of further molecular-level experiments is the fact that H_2_O is fundamentally challenging to study with atomic resolution: The position of the H atoms and the molecular orientation are difficult resolve with imaging techniques[3,7] due to the high mobility and delocalisation of the water protons even at low temperature. Consequently, studies using scanning tunnelling microscopy (STM) are usually restricted to flat metal surfaces[3,6–9,15] and a few ionic crystals, such as NaCl[16,17]. These single crystal studies have evidenced that ice nucleation often happens at surface defects (kink or step sites), where the adsorption energy of water is higher[9,18] (Figure 1). Since graphene became available by the so-called scotch tape technique, a vast class of 2D materials has been investigated on the basis of their electronic properties[19]. Several of these new merging materials, belong to the unifying framework of Dirac materials which exhibit an electronic surface state with a linear energy-momentum relationship, a so-called Dirac cone[20]. In typical Dirac materials such as graphene and topological insulators (TIs), low-energy fermionic excitations behave as massless Dirac particles.

**Figure 2:**
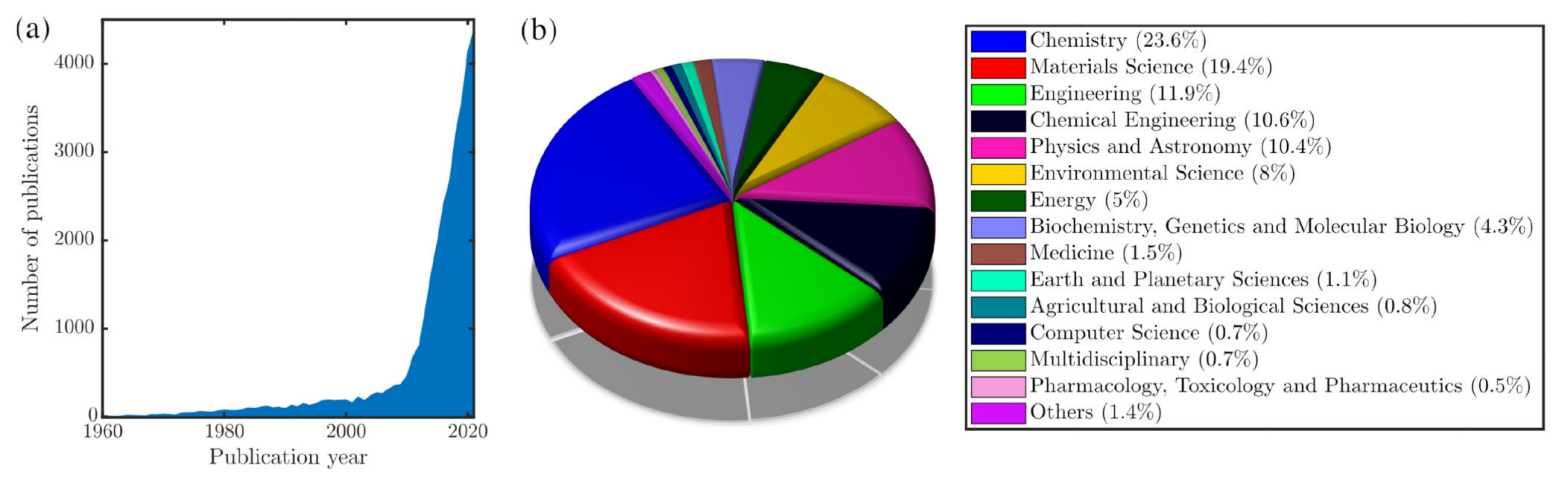
(a) Number of published articles and review articles with “water” and “graphene/graphite” from 1960 to 2021, according to Scopus at the end of March 2022. (b) Distribution according to subject categories.

Apart form their electronic properties and the interest in non-silicon based optoelectronics and photonics devices[21], graphene has emerged as a material to detect and store molecules[22], as separation and desalination membranes[23,24], in nano- and micro-fluidics[25], in electrochemistry and fuel cells[26,27], catalysis[28–30] and as coating for corrosion prevention[31] and anti-icing purposes[32–34]. In addition to these applications, where the water-surface interaction plays a key role, the adsorption of water on graphene has become one of the benchmark systems for modern computational methods, in particular dispersion-corrected density functional theory (DFT). The increasing interest in the topic of water interaction with graphene is best illustrated by the number of publications over the last decades as shown in Figure 2. While at the macroscopic scale an increasing amount of experimental data is becoming available[35], experimental studies providing molecular-level information about water on graphene are relatively new and here “images” with atomic resolution could only be obtained for subsurface water[36] probably due to the high water mobility on graphene. Unravelling the microscopic details of water-surface interaction and dynamics holds implications to the above mentioned applications, e.g. for tailored surfaces but is equally interesting for physicochemical processes in the Earth’s atmosphere[37] as well as astrochemistry occurring on cosmic dust grains[38–42].

**Figure 3:**
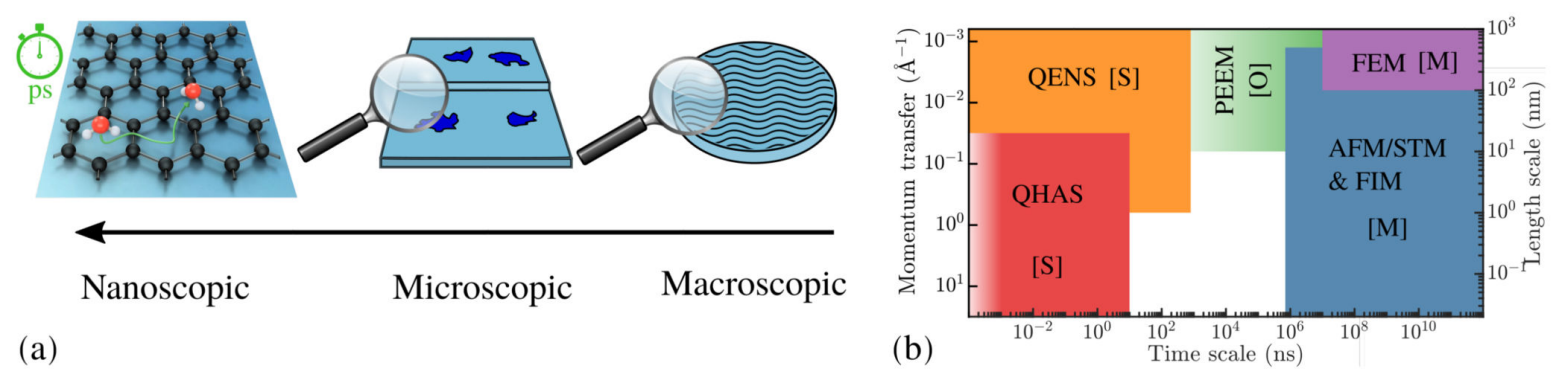
(a) Schematic illustration showing that the interfacial dynamics of water can only be understood at the nanometer length-scale while the relevant timescale for molecular diffusion is the pico- to nanosecond regime. (b) The ps to ns regime is covered with scattering techniques [S] (quasi-elastic neutron and helium scattering: QENS/QHAS), while longer timescales are accessible with scanning probe microscopy [M] (STM/AFM). Optical techniques [O] such as PEEM can provide the temporal resolution via pulse-synchronised methods but are limited in terms of their lateral resolution.

**Interfacial dynamics of water:** Despite water being ubiquitous in everyday life, its nanoscale motion at surfaces and interfaces is far from being understood, in particular since theoretical and computational studies, particularly at low-coverage, suffer from the lack of experimental insight[2,3,6,7,15,17,43–45]. The vibrational dynamics and electronic transitions of water at surfaces as well as the motion of protons, usually occur at ultrafast time scales, in the order of femtoseconds[46]. While these processes are accessible with ultrafast optical spectroscopy [47–49], the interfacial diffusion of molecules as illustrated in Figure 3(a) occurs typically in the pico- to nanosecond regime and is either monitored in real space using microscopic techniques or in reciprocal space using scattering techniques[50–52] as described in section 3 and labelled with [M] and [S] in Figure 3(b), respectively. However, to make these fast diffusive motions accessible to microscopy studies, the process typically needs to be considerably slowed down. At the same time an intrinsic problem of scanning probe microscopy is that the probes inevitably induce perturbation to the fragile water structure, due to the excitation of tunnelling electrons and the tip-water interaction forces[46,53].

In summary, while there exist many studies about the adsorption on graphene and graphite, a molecular level understanding is still clearly missing in order to provide a complete picture which will be discussed in 2. In terms of the dynamics of water, mostly experimental information is missing since the motion of individual water molecules has so far only been reported for a couple of specific substrates as outlined in 3.

### 1.1 Sample preparation

The common feature of graphene and van der Waals (vdW) layered materials is the preparation of thin layers by exfoliation, which also holds for the described layered class of topological insulators. Therefore, the crystal samples can be prepared by *in-situ* cleaving under ultra-high vacuum (UHV) conditions[54]. For graphene, another common method is chemical vapour deposition (CVD) growth of metal-supported graphene *in situ*, with the growth and characterisation of the graphene layer being reported in numerous publications[22]. The graphene results reported here are for a graphene monolayer on Ni(111), grown by dosing ethene (C_2_H_4_) while holding the crystal at 730 K for several hours[55].

Water deposition is done with a microcapillary array beam doser, which can be brought close to the sample surface to reduce the water load in the vacuum chamber. Water adsorption and desorption processes are then studied during dosing with a precise water pressure control obtained by a motorised leak valve, regulated by a feedback control system in order to maintain a constant pressure[56]. Water was supplied to the doser from a baked stainless steel tube filled with de-ionised water, using the vapour pressure over the liquid phase at room temperature. In a typical experiment, water is first purified by pumping and thawing cycles prior to introduction into the vacuum chamber: Water purification follows several freeze-pump-thaw cycles, where the water inside the tube is frozen and the gas phase above the frozen ice is pumped away.

## 2 Water adsorption at graphitic interfaces, nucleation and film growth

**Figure 4:**
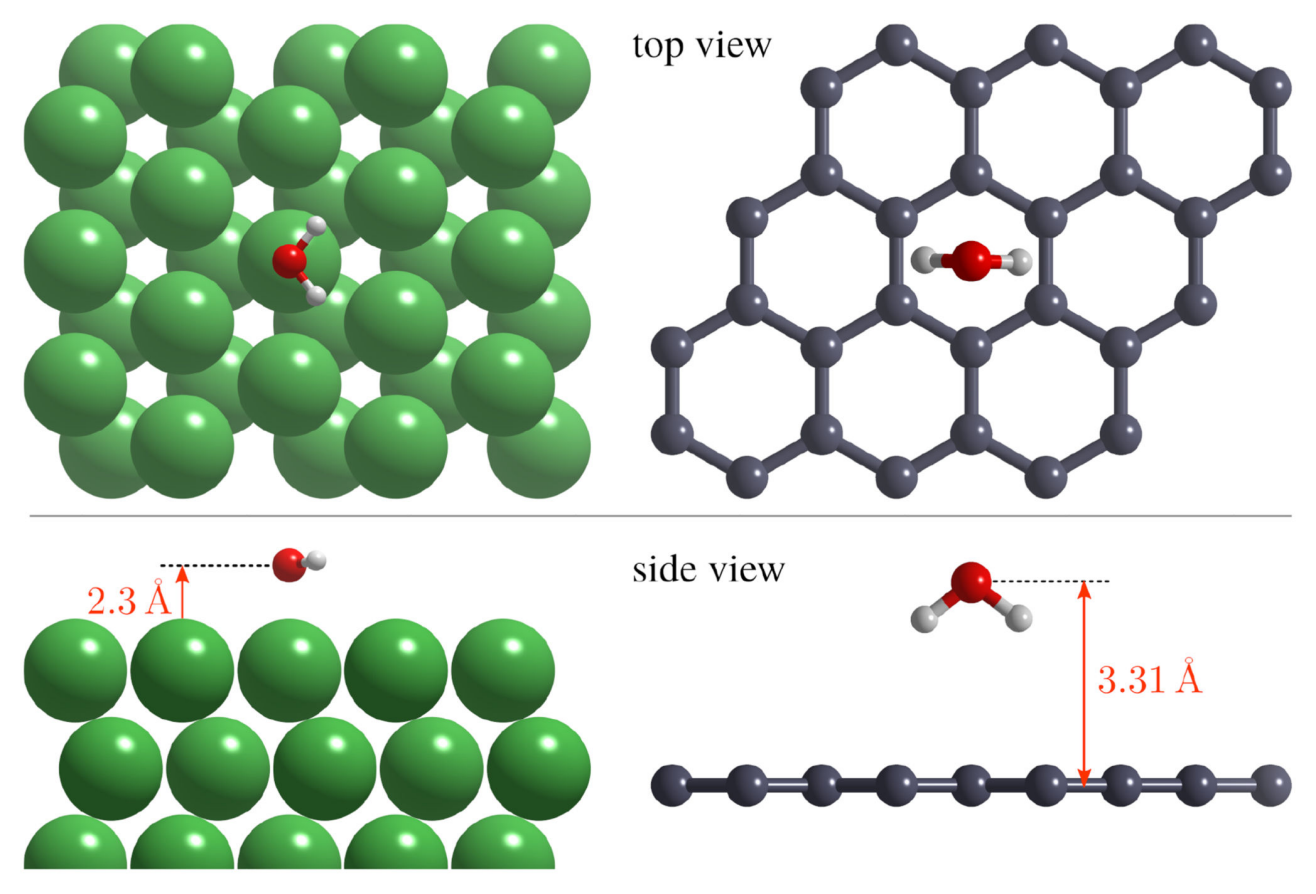
Comparison showing the radically different adsorption geometries for a single water molecule on a metallic surface and graphene. On a close-packed metal surface such as Ru(0001), H_2_O adsorbs with the oxygen atom on top, yielding a higher binding energy and a vertical orientation of the molecule[68], in contrast to the most favourable adsorption geometry on graphene from dispersion corrected DFT[56] with H_2_O in the centre of the hexagon and both O–H bonds pointing towards the surface.

As illustrated in Figure 4, the interaction of water with graphene is fundamentally different to H_2_O/metal systems and we start by describing H_2_O/graphene as a benchmark system for DFT calculations. On metals, water typically forms a bond to the surfaces via the O atom (left panel in Figure 4), and formation of H-bonded clusters is common even at low coverage as the strength of the attractive interaction between 2 molecules is comparable to the substrate-H_2_O bond[6,57,58]. Upon deposition of water at metal surfaces three scenarios are found, partial or complete dissociation of the H_2_O molecule is found, intact wetting (formation of a 2D monolayer), and non-wetting adsorption (forming 3D water clusters and regions of bare metal)[3,6]. For pristine graphene both the adsorption geometry of H_2_O is different (right panel in Figure 4) and adsorption is molecularly rather then dissociatively[9,59]. Moreover, while adsorption probabilities for H_2_O/metal are typically close to unity[57], the initial sticking coefficient of water on graphene is much smaller and tends to decrease with temperature[56].

On the other hand, in terms of film growth similarities with metal substrates Figure 1 can be found. E.g., for highly oriented pyrolytic graphite (HOPG) it was shown that atomic steps induce the aligned growth of ice crystals[60]. Water films grown on HOPG crystallise into a hexagonal monolayer with high density, starting with amorphous water which transforms into ordered layers only after annealing to higher temperatures[61].

### 2.1 Water adsorption on graphene - a benchmark system for DFT

The adsorption of single water molecules on pristine graphene has been one of the most important benchmark systems for DFT calculations for the last decade as the bonding between water and graphene (and other extended conjugated *π* systems such as hBN) is dominated by van der Waals (vdW) dispersion interactions and long-range correlation[62–64]. In 2011, Kysilka *et al.*[65] reported DFT/CC calculations for H_2_O binding energy on graphene and graphite. The authors only considered an adsorption geometry with both O H bonds of H_2_O pointing towards the surface (so called legs-down orientation) and for this orientation the adsorption energies are 12.8 and 14.6 kJ/mol for graphene and graphite respectively.

Ma *et al.*[62] reported for the first time benchmark DFT calculation of H_2_O adsorption on graphene at the RPA level of theory. They also explored 2 different orientations: the one-leg and the two-leg down, with the latter marginally more stable than the former (98 meV compared to 82 meV). An important result of their comprehensive benchmark work was to point out that the widely used BLYP and B3LYP functionals produced purely repulsive binding energies between water and graphene.

A summary of several DFT calculations is shown in Table 1, with he energetically most favourable adsorption site (hollow i.e. at the centre of the graphene hexagon and top) the molecular orientation (down/vertical), the adsorption energies and the adsorption distance. Most calculations predict a preferential adsorption as outlined in Figure 4, with adsorption energies *E_ads_* in the range of about 130 meV. Results of *E_ads_* vary considerably[66,67] while a general agreement on an adsorption distance of ≈ 3.3 Å is observed. In comparison, for H_2_O on transition metals such as Pd(111) or Ru(0001) (Figure 4) much larger binding energies at about 0.3−0.4 eV, exceeding even the water binding energy of ≈ 0.24 eV), and a distance *d* = 2.3 Å are typically found[68].

Furthermore, computationally inexpensive dispersion-correction (DFT-D scheme) perform reasonably well in modelling the water-graphene interactions compared to “pure” GGA functionals[76]. Binding energies of water with a single graphene layer are typically found to be comparable to those of water bound on graphite.However, vdW DFT predicts that for supported graphene, about 30% of the vdW interactions between the water and the substrate are transmitted through graphene[77]. Employing a dispersion-force corrected functional (optB86b-vdW) to calculate the binding of water on free-standing and epitaxial graphene on nickel and copper substrates, for both Ni and Cu, the preferred adsorption site is the centre of the hexagonal cell, marginally (< 10 meV) more stable than bridge and top sites[76]. Again, the preferred orientation of the water monomer is the leg-down, with an equilibrium distance of 3.21 Å for Gr/Ni and 3.31 for Gr/Cu (compared to 3.33 Å for suspended graphene). These theoretical results are in good agreement with XAS measurements by Böttcher *et al.*[74], who reported that the preferential binding site for H_2_O on Gr/Ni(111) is either the centre of an hexagon or the bridge site between the C atoms.

While we do not discuss influences on the electronic structure in detail, it is shortly mentioned that adsorption of water onto graphene causes a shift in the Fermi level of up to 100 meV[75]. For pristine (suspended) graphene the Fermi level shift is negligible while for metal-supported graphene, the overall Fermi level shift strongly depends on the intensity of the graphene/metal interaction. For graphene strongly bound to the metal substrate (e.g., on Ni surfaces), adsorption of water has only a weak effect on the Fermi level position, while for graphene on Cu, the Fermi level shift can reach 100 meV[75].

There exist also several theoretical studies considering larger water clusters, and Water nanodroplets: are further discussed below. E.g., it has been predicted that the binding energies of H_2_O molecules in a cluster are an order of magnitude larger than the binding of clusters to graphene[78]. Desorption from graphene edges has been investigated computationally by Abe *et al.*[79] using MD and DFT methods. The authors report a binding energy on two types of hydrogen-passivated edge sites varying from 0.97 kcal/mol to 1.28 kcal/mol depending on the dimensions of the graphene cluster analysed.

### 2.2 Experimental approaches to water adsorption and structure

#### Helium atom scattering

Neutron diffraction has been extensively used to determine the bulk structures of ice and proton order [80,81], with new bulk structures still being discovered[82]. Helium atom scattering (HAS) is the surface equivalent to that technique, as it is a truly surface sensitive scattering technique and will be shortly described in the following, since it is rather uncommon. A monochromatic He beam can be described as a plane wave, following the wave-particle duality. Since momentum and wavelength are inversely proportional via the de Broglie relation, He atoms with an incident energy of 10 meV exhibit a wavelength of 1.4 Å[83]. Hence scattering from a surface with periodicity on a similar length scale, will give rise to a diffraction pattern - quite analogous to X-ray diffraction - although the scattering mechanism itself is entirely different as the classical turning point for a He atom is several Å above the surface, thus providing excellent surface sensitivity[84].

**Figure 5:**
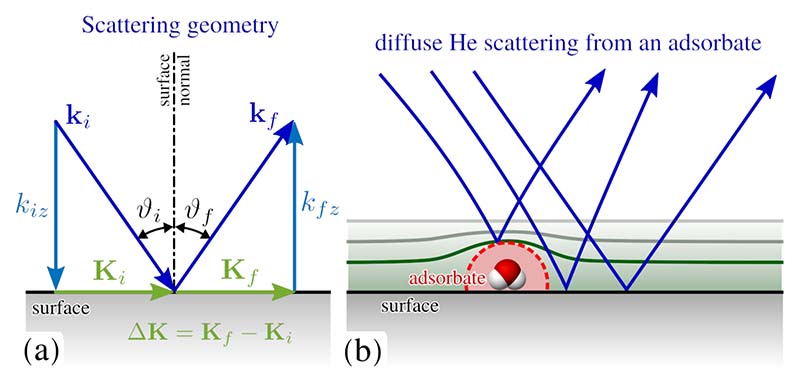
(a) Illustration of the scattering geometry in a HAS experiment, with incident and final wavevectors as **k**_*i*_ and **k**_*f*_, respectively and the incident angle *ϑ_i_* with respect to the surface normal. Components parallel to the surface are given in capital letters, **K**_*i*_ and **K**_*f*_, with the momentum transfer Δ**K** = **K**_*f*_ − **K**_*i*_. (b) Scattering from isolated adsorbates typically gives rise to diffuse scattering as illustrated. Isolated adsorbates exhibit an apparent He scattering cross section much larger than its size as illustrated by the dashed red line. It follows from the scattering process since He atoms are scattered from the electron cloud and the He beam exhibits also “refraction” in the vicinity of the adsorbate as illustrated by the blue lines.

The typical scattering geometry in such a HAS experiment is shown schematically in Figure 5(a). A diffraction pattern is obtained by varying the polar (incident) angle *ϑ_i_* around the corresponding axis while the scattered beam intensity is detected. For elastic scattering, the momentum transfer parallel to the surface, Δ**K**, is given by

(1) $$\left|\Delta \text{K}\right|=\left|{\text{k}}_{\text{i}}\right|\left[\text{sin}\;\vartheta_{f}-\text{sin}\;\vartheta_{i}\right]=\left|{\text{k}}_{\text{i}}\right|\left[\text{sin}\left(\vartheta_{SD}-\vartheta_{i}\right)-\text{sin}\;\vartheta_{i}\right],$$

where **k_i_** is the incident wavevector, *ϑ_i_* the incident angle with respect to the surface normal and *ϑ_SD_* the fixed angle between source and detector.

In addition to diffraction experiments, that reveal information about the surface structure, measurements of the specular He reflectivity can provide information about the degree of order on the surface[83]. The He reflectivity will be greater for a highly ordered surface since less signal is lost in other scattering directions. Hence measuring the proportion of incident He that is scattered into the specular direction is a means of determining the surface quality (step edges and defects). Furthermore, monitoring changes in the specularly reflected intensity upon deposition of atoms and molecules provides also a multitude of information about the adsorption process and growth of films at the surface as shortly outlined below.

**Figure 6:**
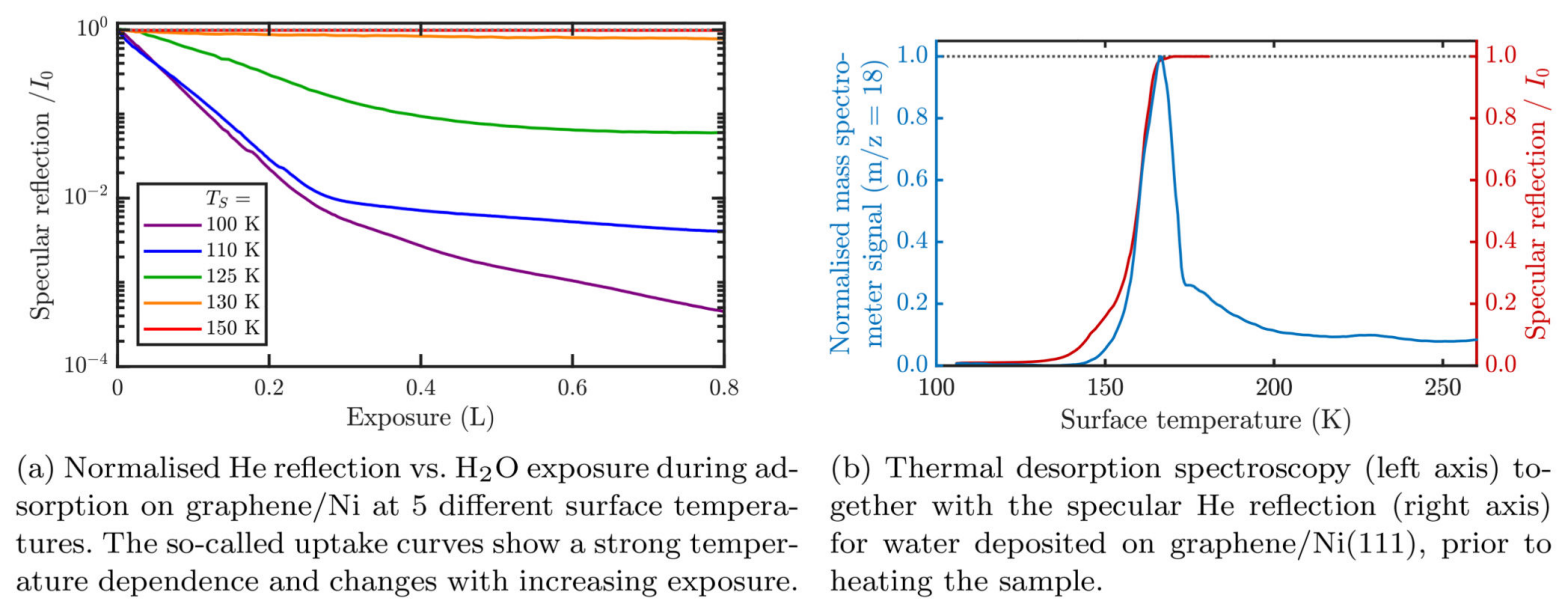
The processes of adsorption and desorption can be monitored by following in real time the specularly scattered He signal during the deposition of adsorbates. Measurements provide coverage calibration, the apparent He scattering cross section Σ and signatures of inter-adsorbate interactions (see text).

The processes of adsorption and desorption can be monitored by following in real time the specular beam intensity of He atoms scattered from the crystal surface during the deposition of adsorbates; the resulting curve is usually called an “uptake” curve[52]. As plotted in Figure 6a, in order to calibrate the coverage and to investigate the adsorption processes, the He revflectivity *I* is measured while dosing or evaporating at fixed surface temperatures. In the low coverage limit, the He scattering cross section, Σ, for isolated adsorbates can be calculated as $\sum ={-\frac{1}{n_{s}}\cdot \frac{1}{I_{0}}\cdot \frac{\text{d}I}{\text{d}\Theta }|}_{\Theta =0}$ where Θ is the coverage given by the number of adsorbates per substrate atom, *n_s_* is the number of substrate atoms per unit area, and *I/I*_0_ is the specular helium beam attenuation at coverage Θ[83]. As illustrated schematically in Figure 5(b), the apparent He scattering cross section, Σ, for isolated adsorbates is often much larger than the adsorbate size due to the scattering process and refractive effects in the vicinity of the adsorbate. Values for small molecules are often in the range of several 100 Å^2^, e.g. for water on graphene/Ni(111) a cross section of Σ = 120 Å^2^ was found[56].

The fact that the presence of adsorbates on the surface substantially attenuates the specular beam can be used as a measure of adsorbate coverage. Uptake curves are useful to determine the coverage or to study the lateral interaction between adsorbates and to determine when regular overlayer structures occur[83]. For non-interacting adsorbates occupying random adsorption sites with large cross sections that overlap, the specular attenuation as a function of coverage is linear on a logarithmic scale[83].

**Figure 7:**
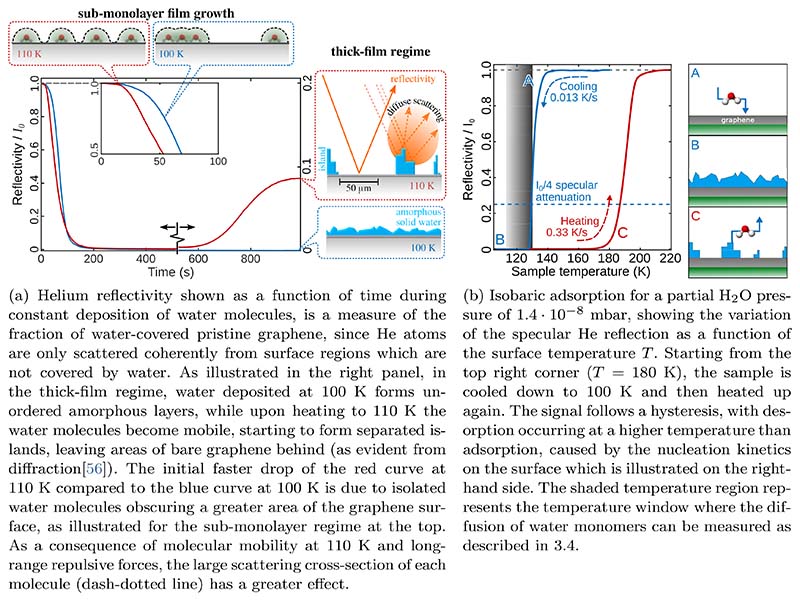
Adsorption and island formation of water on graphene from helium scattering measurements.

The overlap of giant cross sections with increasing coverage, provides also information about the adsorbate interatomic forces [83]. Repulsive forces result in adsorption sites being further apart (top panel of Figure 7a), the scattering cross sections of the individual adsorbates overlap less compared to the non-interacting case, and thus the uptake curve falls below the linear line. Attractive interactions on the other hand, will give rise to a larger overlap and the curve rises above linear.

**Scanning-probe techniques**, such as STM have been invaluable in the determination of local atomic water structures by real-space images of water monomers via small clusters to monolayers[3,6–9,15]. Low-temperature STM is often combined with DFT calculations, as the interpretation of topographic STM measurements is not always straightforward considering details such as the orientation of water molecules and the differentiation between molecules and OH species. Moreover, the tunnelling parameters need to be carefully chosen, as the tip may disturb the water molecules or influence their bonding structure[46,53]. On the other hand, tunnelling electrons can also be used to intentionally excite, restructure, and dissociate water molecules[1]. Finally, non-contact atomic force microscopy (AFM) has recently also been successfully employed to determine the structure of thicker water films[85].

#### Complementary experimental techniques

In terms of experimental techniques, we limit this short review to HAS and STM, as those are the only techniques currently able to measure the atomic-scale dynamics of water at surfaces (see Figure 3(a) and 3.2). We note that of course many surface science techniques have been used to study the water-surface interaction providing thus invaluable insight due to their complementary information. Many early structural results go back to low energy electron diffraction (LEED), with the implicit risk of inducing dissociation and to some extent also desorption[3,7].

X-ray photoelectron spectroscopy (XPS) and X-ray adsorption spectroscopy (XAS) provide chemical information about the adsorbed water molecules, in particular when combined with temperature-programmed desorption spectroscopy. Similar to LEED, XAS and XPS have the potential to produce damage, via photodissociations or by means of secondary photoelectrons[1]. In contrast, HAS as outlined above, is a non-destructive technique which is sensitive to the position and ordering of hydrogen atoms and has been successfully used to study the termination of thick ice films[86]. Vibrational techniques, such as high-resolution electron energy loss spectroscopy (HREELS), infrared (IR), and sum frequency generation (SFG) spectroscopies can be used to study hydrogen bonding and may help determine the orientation of the bonds[1].

In summary, HAS has been used to determine the termination of thick ice layers grown on metal substrates[86]. Because of the mentioned large scattering cross section of HAS to isolated adsorbates, in particular hydrogen and H_2_O, the position and structure of hydrogen atoms and adsorbed water layers can be readily determined[45,53,87–89] while H-positions are hard to determine with other methods (e.g., hydrogen is a weak scatterer for electrons) which also present a severe risk of damaging the H-layer[7,90]. For example, in a study of highly proton-ordered water structures on oxygen pre-covered Ru(0001) it could be shown that the atomic oxygen and the oxygen from water forms a (2 × 2) surface reconstruction, which however, is broken by the hydrogen to give a (2 × 4) surface reconstruction: LEED measured a (2 × 2) pattern, while HAS measured a (2 ×4) structure[91]. Moreover, HAS can be used to probe the proton order[45] and ice formation at highly corrugated adsorption templates[53].

### 2.3 Adsorption on graphene and bulk graphite

While a short section on sample preparation and the deposition of water deposition can be found in section 1.1, we discuss now the adsorption behaviour on graphene, based on a recent HAS study [56] and compare the findings to other experimental accounts for graphene and bulk graphite. Figure 7a shows an above described “uptake curve” for water adsorption on graphene/Ni(111). The intensity of the reflected He beam is monitored, while the cold graphene surface is exposed to an increasing amount of water, from sub-monolayer up to the thick-film regime. The reflected intensity comes from the graphene surface, while water or ice forms a disordered arrangement and thus almost no reflected intensity is registered from ice-covered areas. Preparing thick water films with the graphene surface at 100 K this way, completely suppresses the helium reflectivity (right panel of 7a). However, upon heating the surface, beyond a certain temperature the reflected intensity recovers after some time: The ice-covered areas become smaller, leaving the graphene substrate behind. Based on the fact that exactly the same diffraction pattern is observed as from clean graphene[56], upon heating the surface the increased mobility of the water molecules gives rise to a kind of “de-wetting” process, leaving areas of bare graphene between separated islands of ice behind (right panel of 7a).

The low temperature behaviour, i.e. the formation of amorphous ice layers on surfaces, commonly referred to as amorphous solid water (ASW) has been observed since the 1960s[92]. Even on metal surfaces, due to the growth kinetics and reduced mobility of the water molecules, below a certain temperature ASW films can be observed[93,94]. Similarly, for water on HOPG it was reported that water adsorbed at low temperature is not incorporated into a crystalline surface due to kinetic limitations, and the surface layer exhibits an amorphous character[95].

Although the different temperature or coverage regimes of specific studies cannot always be easily compared, similar results in terms of the 3D islands and de-wetting with increasing temperature have been reported both for bulk graphite and metal-supported graphene. For example, on graphite[96] there is evidence that ice with a thickness of hundreds of monolayers coexists with regions of bare graphite, similar to the described situation for graphene/Ni(111). Isothermal desorption measurements of water on HOPG at 100 K, showed a glass transition accompanied by a change in desorption rate and a growth of 3D water islands, rather than a wetting of the graphite surface[97]. Finally for water on graphene/Pt(111), ASW was reported at low temperature, while above 140 K non-wetting, three-dimensional ices are formed[98].

Further information about the adsorption behaviour can be obtained in another kind of experiment: Figure 7b shows an isobaric deposition curve of water on the graphene/Ni(111) surface where at a constant partial pressure of H_2_O the temperature of the crystal is decreased from 180 K down to 100 K. There is no significant decrease in the He reflectivity until the crystal reaches about 140 K where the intensity of the specular peak falls off sharply corresponding to the commencement of adsorption. The specular intensity drops to almost zero when the crystal temperature has reached 100 K. Upon starting to heat the system under the same conditions the reflectivity does not increase before reaching temperatures above 160 K, showing a hysteresis i.e. desorption occurring at a higher temperature than adsorption. The hysteresis shows that there exists a kinetic barrier to nucleation on the surface, with the molecular nature of this barrier being discussed later in the dynamics section 3.4. As illustrated by the cartoons in the right panel of 7b, adsorption on the hydrophobic bare graphene surface[99,100] is less likely before clustering centres start to evolve[74] and starts to set in at much lower temperatures. On the other hand, upon heating, the surface is already covered with amorphous ice, from which it is harder to remove a molecule and hence the intensity only starts to recover at about 160 K.

Here similar results have again been found for graphene: For graphene on metal substrates where a Moiré superstructure with a periodic height variation of the graphene layer forms, it has been reported that the regions closest to the metal substrate act as nucleation centres. In STM measurements extended arrays of amorphous water clusters form on epitaxial Gr/Ir(111) is found[101].

A “classic” yet still very useful surface science experiment is of course thermal desorption spectroscopy (TDS) after water films have been deposited at the corresponding surface. Several groups have conducted TDS measurements of water on the (0001) basal plane of graphite, reporting a single desorption peak at desorption energies in the range of 0.4 – 0.5 eV[102–105], quite close to the sublimation enthalpy of ice at 0 K, 0.49 eV[102]. Since it was observed that the desorption energy does not change with coverage it indicates again a de-wetting of the graphite surface[105].

Ideally desorption measurements following the specularly reflected He signal as described above are conducted simultaneously with TDS where the m/z ratio on a mass spectrometer is measured. Figure 6b shows a typical thermal desorption measurement after the preparation of a thick ASW film on graphene/Ni(111). Clearly visible is one dominant peak with a maximum in the TDS spectrum which coincides with a rapid recovery of the specular signal (right axis in 6b) and corresponds to a desorption energy *E_des_* = 0.52 eV according to the Redhead equation[56]. The desorption energies are thus all within a similar energy region, both for water on metal supported graphene as well as for graphite[96,106,107]. One exception is the study of Standop *et al.* which reports that for amorphous water clusters on the Moiré of Gr/Ir(111), water desorption from the cluster with an energy of 424 meV is about 100 meV lower than desorption of water from ice grown on the same surface.

We close this section by mentioning that water may also intercalate in the case of metal-supported graphene layers which will be discussed in the dynamics part 3.1. As found in STM studies water attacks line defects on graphene/Ru(0001) opening a pathway for water intercalation[108] and reports for Gr/Ni(111) mention that water molecules intercalate below Gr and partly dissociate on Ni(111)[109]. Much of this behaviour is down to the existence of defects [110] and is not observed in the case of defect-free graphene[109] as also confirmed by water adsorption / desorption being a completely reversible process[56].

#### Wettability

On the macroscopic scale, the wettability of graphene is often determined using methods such as contact angle measurements[111,112]. Several theoretical and experimental studies have demonstrated that graphene possesses so-called wetting “transparency” or “translucency”. Essentially, the wetting behaviour of graphene adsorbed on substrates can be similar to the underlying substrate (wetting transparency) or similar to that of suspended (or “free-standing”) graphene (wetting opaqueness) depending on how strong the hydrophilicity or hydrophobicity of the substrate is; if the substrate is highly hydrophilic, the hydrophilicity of the substrate will “transmit” to the graphene monolayer. On the contrary, if the underlying substrate exhibits highly hydrophobic character, the epitaxial graphene will show so-called “wetting opaqueness” and water nanodroplets will have an almost identical contact angle to that of suspended graphene. This substrate-dependent behaviour of graphene is denoted in the literature as having “wetting translucency” and it is explained by the varying intensity of the dispersion interactions between the metal and the honeycomb carbon lattice[77,113,114]. Independently from the substrate, the equilibrium contact angle of water droplets on graphene is weakly dependent on the size of the nanodroplet[113]. The conflicting accounts as to whether graphene is hydrophobic or hydrophilic found in experiments[115] is likely caused by different experimental conditions, i.e. vacuum vs. environmental measurements as well as the quality of the graphene layer i.e. the above mentioned influence of graphene defects in nucleation[110].

### 2.4 Water on novel / 2D materials

**Figure 8:**
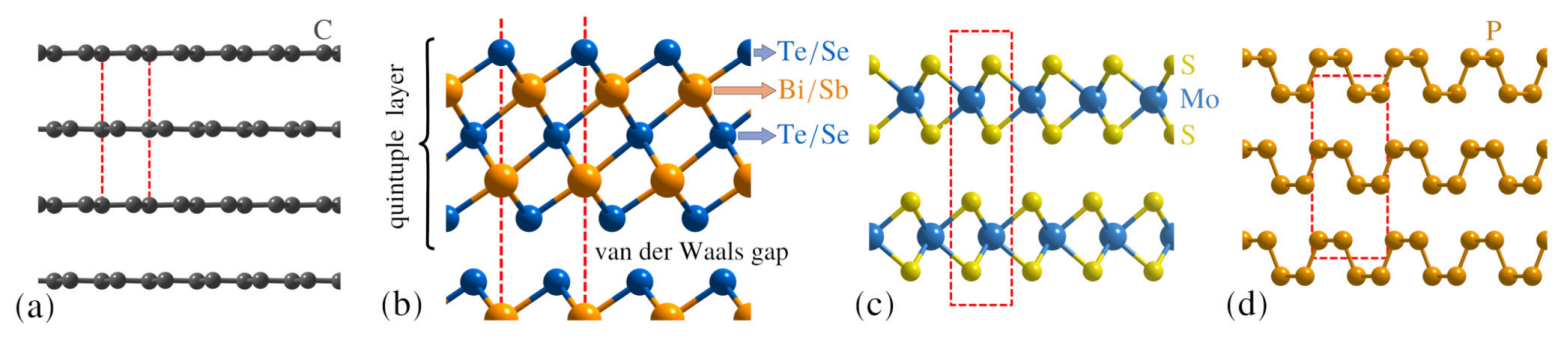
Many novel / 2D materials show a typical layered structure, with intralayer bonding being mostly covalent, whereas the layers are held together by weaker interactions predominantly of vdW character. (a) Layered structure of graphite, with individual layers being graphene. (b) The binary topological insulators are composed of quintuple layers with the terminating layer being either Te or Se. The hexagonal unit cell (which continues to the top and the bottom) is illustrated by red dashed lines. (c) The transition metal dichalsogenide MoS_2_. (d) Black phosphorus.

We discuss now some recent advances in terms of water adsorption and dynamics on novel and 2D materials beyond graphene[116]. The well-known physics and chemistry of 3D bulk matter often become irrelevant for 2D materials, and first attempts to employ exotic phenomena in vdW layered crystals[19] via vdW heterostructures and devices can be found in Ref[117]. Among the most prominent 2D materials, despite the semimetal graphene, are the insulator hexagonal boron nitride (h-BN) and the transition metal dichalcogenides (TMdCs) which tend to be semiconductors (Figure 8(c)). A recent review about the interaction of 2D materials with water can be found in Ref. [118], which concentrates however mostly on the macroscopic rather than molecular level using e.g. contact angle measurements. Similar for TMdCs such as MoS_2_ the water-surface interaction is mostly studied in terms of wetting behaviour [119–122] with few molecular level approaches [119].

#### TIs

For a start we will consider the layered material class of TIs such as Bi_2_Te_2_, which together with graphene belong to the class of Dirac materials. Surface chemistry and the water-surface interaction on these materials is of interest for sensing applications[123] and unique properties upon exfoliation in liquid environments[124]. From angular resolved photoemission spectroscopy it was reported that water adsorption on Bi_2_Se_3_ gives rise to an *n*-doping of the surface[125] and a reaction of Bi_2_Te_3_ with water was found in STM works[126]. However, adsorption of water on Bi_2_Te_3_ is negligible, at least at room temperature[52,127] and thus the aforementioned results could simply be caused by the probing technique as e.g. doping upon adsorption is in-fact often caused by illumination-triggered photoionisation and -dissociation[128]. The latter may of course be interesting for perspectives of photo-catalytic water splitting[129,130], also in the context with TMdCs[131].

The H_2_O/Bi_2_Te_2_ system shares some similarities with graphene, both in terms of “wetting” and the adsorption kinetics. At low temperature ASW is observed confirmed by the lack of any HAS diffraction pattern[52]. Water adsorption/desorption on Bi_2_Te_3_ is again a completely reversible process, as confirmed by He reflectively and diffraction measurements that exclude any structural changes. Moreover, with increasing temperature the sticking coefficient decreases significantly[52], in line with the reported negligible surface reactivity of Bi_2_Te_3_[127].

#### h-BN

Hexagonal boron nitride (h-BN) is a 2D material with an honeycomb structure identical to graphene, but with heteroatomic B-N bonds instead of C-C bonds[132]. Contrary to C-C bonds, B-N bonds have a partially ionic nature and as a consequence h-BN exhibits a much wider band gap (5.95 eV) than graphene and is therefore an insulating material. The bonding between water and h-BN and the wettability of flat h-BN has been the subject of recent theoretical and experimental studies[63,133–135]. Wu and co-authors[63] applied Quantum Monte Carlo (QMC) and several others quantum chemistry methods to calculate the binding energy of water on h-BN. Their results show that Møller-Plesset perturbation theory (MP2) is accurate for this system and they used their MP2 results to fit force field parameters for modelling h-BN - water interactions, while dispersion-corrected DFT tends to overestimate the binding energy of water similar to graphene. The ice structure on metal-supported h-BN with the h-BN overlayer forming a Moiré[136], was studied with STM[137] and first principles calculations [138] and shows similarities to water on a graphene Moiré as described above. In general there exist surprisingly little experimental studies about water on h-BN. In terms of water dynamics, Tocci *et al.* predict a substantially larger macroscopic friction coefficient compared to graphene from MD simulations[139].

**Black phosphorous** is a stable allotrope of phosophorous[140] (Figure 8(d)) and has, in the last decade, been studied as one one the most interesting among the emerging 2D materials[141,142]. Since it is a semiconductor with a tunable bandgap 2 eV and an extremely high carrier mobility, it has been applied for manufacturing of electronic and optoelectronic devices, including transistors and photodetectors. The wetting of black phosphorous (BP) was studied by Zhao and co-authors[140] by optical microscopy showing that water forms elliptical droplets on the anisotropic BP surface compared to rounded droplets on graphene and MoS_2_.

## 3 Interfacial dynamics of water

The interfacial motion of molecules remains a central question to fields as diverse as catalysis, friction, crystal growth and ice nucleation. However, as already mentioned in the introduction, much of our existing knowledge concerning the microscopic motion comes from computational simulation[139,143]. In early experimental works, the focus of many techniques was to deduce diffusion constants and make a connection to macroscopic diffusion theory[50,144]. However, only on atomic length-scales the underlying physical behaviour of the diffusion process is revealed. Moreover, only at elevated temperatures, when atoms and molecules move on fast timescales many complicated forms of diffusive motion emerge. Thus, the study of surface diffusion processes is a unique and challenging problem for experiments, as it requires both sub-nanometer spatial resolution and fast (pico- to nanosecond) temporal resolution as outlined in the following[50]. Consequently, there exists still relatively little experimental knowledge about the fundamental nature of diffusive processes at the molecular level. In the following we first discuss some recent findings about confined diffusion of water and water nanodroplets before we describe the measurement principle and recent results concerning the single-molecule diffusion of water.

### 3.1 Confined diffusion in graphene nanochannels and intercalated water

In the last decade, several studies demonstrated the uniqueness of confined water dynamics, including diffusion and self-assembly, in confined systems[145]. Similar to the “new” structures of water found upon deposition on a surface, nanoconfined water may crystallise into a plethora of novel ices never seen in bulk water[146,147]. These confined environments are of particular technological importance for electrochemistry, water desalination[24], filtration[148], biological[149] and biomedical applications[150]. Here we limit ourselves to review the most recent studies that have addressed the atomistic behaviour of confined water in graphene. Theoretical and experimental studies reported that water dynamics strongly depends on the surface curvature, the thickness of the graphene layers, the mechanical pressure and the spacing between those[151–169].

Yang and Guo reported an MD study in which they found evidence than the friction of water diffusing in graphene nanochannels depends also on the chirality of the layers contacting the water, with larger friction for armchair orientation compared to the and zigzag orientation[161]. Stretching effects on water dynamics were analysed by Wu *et al.* by MD simulations in which the authors varied the amount of mechanical stretching in the water layer for three channel widths. Their results show that stretching (or negative pressure) causes an increase in the diffusion rate of water in the nanochannels[160]. Qiao *et al.* studied the hopping mechanism of water confided in graphene nanochannels by MD, i.e. jumping between the lower and upper graphene layer in contrast to lateral diffusion along the layer, where they observed that local fluctuations of the density and hydrogen bond configuration drive an activated jumping process.

Further information about the dynamic properties of nanoconfined or intercalated water often comes from AFM measurements, which is typically concerned with mechanic properties rather than molecular level details. E.g., using AFM it was shown that the mechanical properties of nanoconfined water layers change significantly with their dynamic state[170]. For H_2_O intercalated between graphene and mica it was found that friction increases by a factor of ≈ 3 relative to dry mica[171]. Moreover, the friction between the tip and the substrate increases, depending on the thickness of the water and graphene layers[172]. Finally, the effect of humidity and water intercalation on the tribology of few-layers of graphene and graphene oxide has been reported by Arif and co-authors[173].

#### Water nanodroplets

The graphene capacity of assuming hydrophobic and hydrophilic characteristics has been recently exploited for creating unidirectional water transport channels by varying the graphene sheet pattern[174–177]. These channels can control the flow of liquids at the nanoscale[174] and can be employed in biomedical devices for fast and accurate drug delivery and in engineering for enhanced heat transport and even for generating electricity[178,179]. From the increasing amount of studies considering water nanodroplets on graphene [44,101,180–182], we only mention a few findings below.

Seki and co-authors[174] investigated the effect of the number of layers of graphene on the adsorption and diffusion properties of water. The hydrophobicity of the graphene surface is highest for single-layer graphene and decreases as a function of the number of layers. The water adsorption energy was lower on the single-layer graphene compared to the triple-layer and six-layer graphene. Finally, they show that the hydrophobic character of the surface decreases as the water coverage increases.

Papadopoulou and co-authors demonstrated unidirectional pumpless transport of water nanodroplets on patterned graphene, with nanodroplets’ speed exceeding 100 m/s. MD simulations performed by the authors show that the high diffusion rates reached by the droplets are due to contact angle hysteresis and depend on the surface pattern and the droplet size. The ultrafast motion of water nanodroplets on graphene cones was investigated by Zhang *et al.*[115], who found that the droplets go through different phases of acceleration and deceleration during diffusion and assessed the influence of droplet size and apex angle of the cone on droplet speed.

### 3.2 Experimental approaches to single-molecule diffusion

Several review articles and books provide an overview of experimental methods used to measure surface diffusion at atomic length scales[50,144]. Generally, imaging techniques occupy the longer timescales while scattering methods such as quasi-elastic neutron scattering (QENS)[183–185] and quasi-elastic helium atom scattering (QHAS)[50–52] offer greater potential in the short timescale region as described below. Microscopic techniques such as STM are attractive for their simplicity of analysis. The simplest method is to correlate successive “frames” of static data into a video of the motion - a method which is also referred to as “video”-STM.

The fundamental difference between real and reciprocal space (scattering) techniques lies in the way in which the space and time averaging over the measurements occurs. In the correlation of real-space techniques averaging occurs either over long trajectories or longer times. Hence the real space nature of STM measurements provides valuable insights into the dynamics but the fact that the motion is probed through snapshots does not allow to obtain detailed information about the path of the motion. For experiments utilising scattering techniques, averaging is done in reciprocal space over the whole experiment to increase the signal, while the details of the corresponding motion in real space are maintained. Hence while scattering techniques are more difficult to analyse than their real space counterparts, since they reveal indirect information in reciprocal space, they convey the full breadth of microscopic detail[50].

#### Adsorbate dynamics and diffusion in scattering experiments

**Figure 9:**
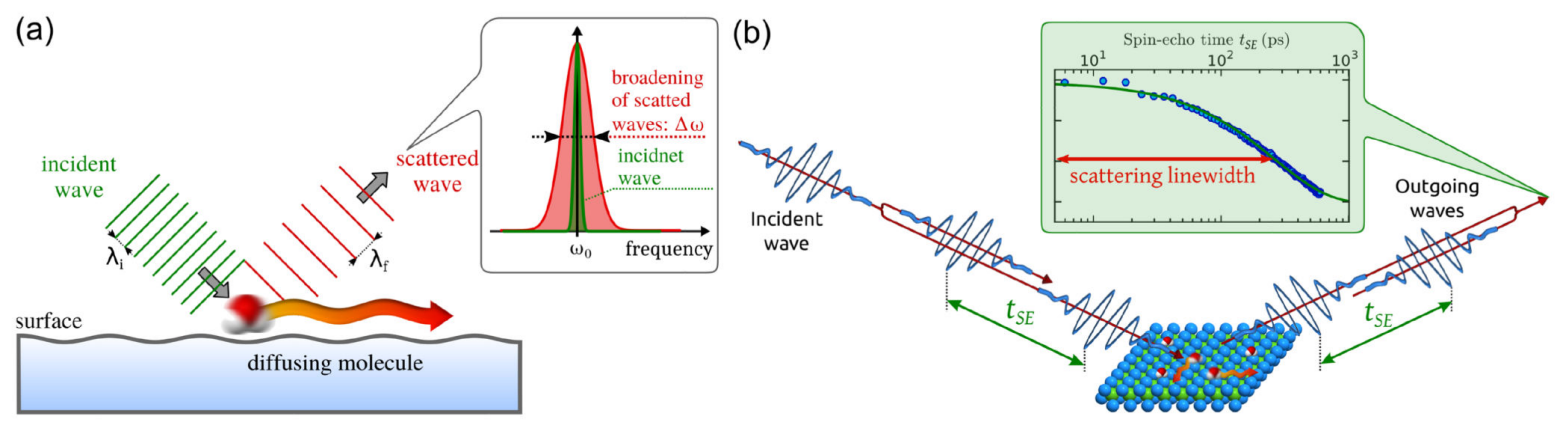
(a) Illustration of the linewidth broadening due to scattering of a plane wave from a moving molecule. The frequency of the incident wave changes upon scattering from the moving molecule (water), in analogy to the Doppler effect (exaggerated by the distance between the wavefronts). The wavelength distribution of the scattered waves is broadened (Δ*ω*) with respect to the nearly monochromatic incident wave with *ω*_0_. (b) Movement on a sample surface can be probed by scattering two wavepackets, spread by a time delay *t_SE_*. Upon recombination of the two scattered wavepackets a loss in correlation is measured due to a small Doppler broadening when scattering from moving adsorbates. The measured ISF shows an exponential decay in spin polarisation with *t_SE_*, from which the decay constant (scattering linewidth) is obtained.

Scattering a monochromatic particle beam from a mobile adsorbate, causes energy exchange similar to inelastic scattering. However, in contrast to phonon events with hω, energy changes of the scattered waves are much smaller ($\underset{\approx }{<}$ meV), occurring over a range of different energies centred around the elastic process (Δ*E* = 0) thus giving rise to a broadening around the elastically scattered peak. Therefore, the process is named quasi-elastic scattering and for HAS acronymised as QHAS. The effect is analogous to Doppler broadening in atomic physics, where the spectral lines of atoms and molecules in the gas phase are broadened due to the Doppler effect caused by the velocities of the moving atoms. In Figure 9(b) the process is shown in a simple illustration. Scattering of a plane wave from a moving adsorbate, gives rise to a change of the wavelength or frequency. As the moving adsorbates exhibit a distribution of velocities, it causes a broadening in the frequency distribution of the scattered waves as shown in the inset of Figure 9(a), in comparison with the nearly monochromatic incoming wave at a central frequency *ω*_0_. The broadening due to the molecular motion is small but measurable and the specific shape of the broadening is determined by the diffusion mechanism and the rate of movement. Hence studying the broadening gives access to the diffusive motion itself.

#### The spin-echo principle

Helium spin-echo (HeSE), as illustrated in Figure 9(b), probes the diffusion of adsorbates by detecting the described small Doppler broadening upon scattering from moving adsorbates. Essentially, the method uses Larmor precession of the nuclear spin of He atoms as an internal timer on each individual particle in the beam. Using ^3^He, any energy change is converted to a loss of spin polarisation: The nuclear spin state of the incident ^3^He is polarised and split into two coherent wave packets, which reach the sample with a time delay *t_SE_*, the so-called spin-echo time (Figure 9(b)). After scattering, the wave packets are recombined and the resulting polarisation is measured. In the case of dynamic processes on the surface, the wave packets scatter differently and, once recombined, a reduced final beam polarisation is found. As the process is based on self-interference of each ^3^He atom, the polarisation loss depends only on the change in energy and not the beam energy itself, resolving energy changes that are as small as 20 neV[50]. Because the impinging He atoms have very low kinetic energies (< 10 meV), they do not affect the observed motion, while at the same time the large He atom scattering cross section provides an outstanding sensitivity[52,186].

Spin-echo measurements provide direct access to the intermediate scattering function (ISF) *I* (Δ**K***, t*), which is directly related to the van Hove pair correlation function[183]. *I* (Δ**K***, t*) is a measure of correlation after time *t* = *t_SE_*, on the length-scale and direction given by Δ**K**. Both variables are adjustable in a spin-echo experiment: Δ**K** is given by the incident beam energy and the scattering geometry (Figure 5(a)) and *t_SE_*, is determined by the spin manipulation applied in the spin-echo coils, which can be varied by adjusting the current in the winding of the coils[187]. The “dephasing” rate *α*, illustrated as scattering linewidth in Figure 9(b) is a measure for the loss of correlation with time due to the diffusive motion. The signature of different diffusive regimes is contained in the dependence of the dephasing rate *α* of the ISF on the momentum transfer Δ**K**, as described in the following section.

Since HAS is inherently coherent, the ISF of a HeSE measurement will be composed of a self-diffusive part and a collective diffusion part, with the latter being responsible for correlation effects if adsorbate interactions become significant at higher coverages[50,144]. On the one hand, HeSE includes the effects of both single-particle and collective diffusion (adsorbate–adsorbate interactions), on the other hand, separation of these two parts is not always straightforward in contrast to neutron scattering, where sometimes tuning via deuterated/hydrogenated isotopes which scatter coherently/incoherently is possible.[183–185]. Nevertheless, as described in further detail in Ref.[188], using an approximate scattering form factor and estimates of the quasi-elastic structure factor allows to obtain the result for incoherent scattering and the corresponding single-particle dephasing rate.

### 3.3 A single molecular perspective to water dynamics

For sufficiently low temperatures or large diffusion barriers, surface diffusion will be dominated by the periodic arrangement of the surface atoms and the molecular motion occurs as discrete hops or jumps between preferred adsorption sites. Based on the analysis of neutron scattering data from 3D liquids[189], an analytic model can be adapted to describe the hopping of adsorbates on surfaces as Chudley-Elliott (CE) model[50,144]. It assumes that an adsorbate instantaneously jumps from one adsorption site to the other, with the probability *p_n_* = 1/*τ_n_* (see Figure 12b). Starting point is the rate equation[183,190]

(2) $$\frac{\partial }{\partial t}G_{S}\;\left(\text{R},t\right)=\frac{\text{l}}{N}\sum_{n}p_{n}\;\left[G_{S}\;\left(\text{R}+{\text{l}}_{n},t\right)-G_{S}\left(\text{R},t\right)\right],$$

where **l**_*n*_ are the jump vectors and *p_n_* is the probability that a jump to the corresponding site occurs. Based on the Fourier relations and assuming that hopping occurs between adsorption sites that form a Bravais lattice, it follows that the ISF is an exponentially decaying function according to *I* (Δ**K**, *t*) = exp [−*α* (Δ**K**)|*t*|]. The dephasing rate *α* (Δ**K**) exhibits then the functional dependence in terms of Δ**K**:[50,52]

(3) $$\alpha \left(\Delta \text{K}\right)=\frac{2}{\tau }\sum_{n}p_{n}\;{\text{sin}}^{2}\;\left(\frac{\Delta \text{K}\cdot {\text{l}}_{n}}{2}\right).$$

According to (3), the dephasing rate *α*(Δ**K**) follows the typical sin^2^ dependence versus Δ**K** as illustrated by the green line in Figure 11(a). For any momentum transfer Δ**K** that corresponds to multiples of the lattice spacing in real space (2*π*/*a* - i.e. the Bragg diffraction peaks for the substrate), the ISF remains constant as a function of time *t*, while in between it decays quickly. The amplitude of the sinusoidal shape according to (3) is given by $\frac{1}{\tau }$, with *τ* being the mean residence time between motion from one adsorption site to the other. Even when a number of different jump lengths **l**_*n*_ in (3) are possible, the minima of *α*(Δ**K**) will still be at the Bragg peak positions of the substrate lattice. The CE model contains also Brownian diffusion as a long range diffusion limit, i.e. for Δ*K* → 0 the broadening converges to a parabola[185] and thus approaches the same Δ**K** dependence as for Brownian motion.

Finally, the diffusion coefficient *D* for 2D motion along a particular surface direction (given by Δ**K**), can then be calculated from the hopping rate as determined from the CE model using:[50,52,191]

(4) $$D=\frac{1}{4}{\langle l\rangle }^{2}ϒ$$

where ϒ is the hopping rate and 〈*l*〉 is the mean jump length.

#### Energy dissipation

**Figure 10:**
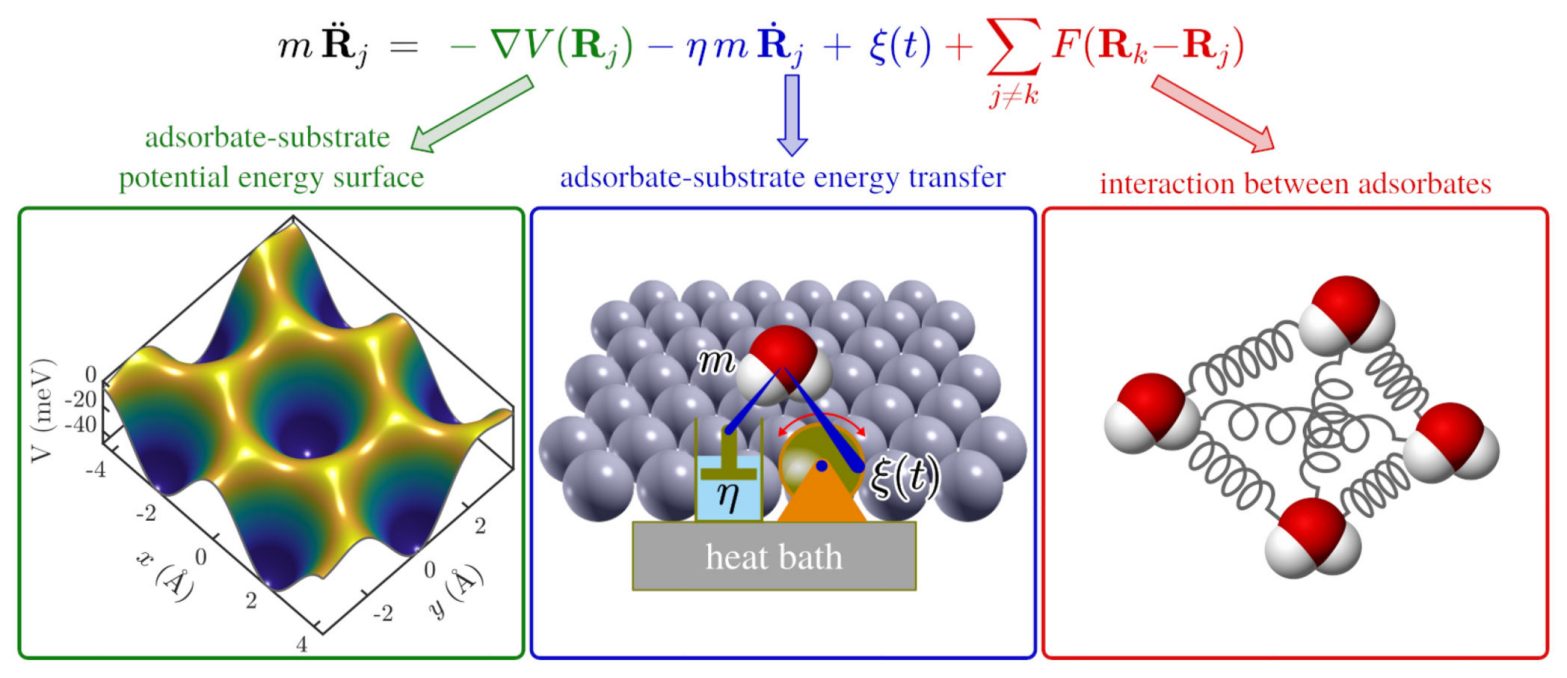
Schematic illustration of the three components in the Langevin description of surface diffusion: (i) a (static) adsorbate-substrate potential energy surface, (ii) adsorbate-substrate coupling in terms of the rate of energy transfer (friction *η* and excitations *ξ*(*t*))) and (iii) pairwise interactions between the adsorbates.

A general approach which considers energy dissipation between the diffusing adsorbate and the substrate together with vibrational motion of the molecules, is provided by the Langevin description of dynamics[50,144,191]. Therefore, the interaction between the adsorbate and the large number of atoms in the surface is approximated by a “frozen” lateral potential energy surface (PES) *V* (*x, y*) = *V* (**R**)[50] with friction being a direct measure of the coupling between the centre-of-mass motion and the heat-bath of the substrate[190]. As illustrated in Figure 10 the forces acting on the adsorbate can be separated into two classes, namely adsorbate-substrate and adsorbate-adsorbate interactions.

While rapid motion represented as a “thermal heat bath” is “averaged”, slower translational motion as “seen” in the experiment is given explicitly by the particle coordinates **R**_*j*_. Adsorbate-substrate interactions are determined by the gradient of the adsorbate-surface interaction potential *V* (**R**_*j*_) and the friction coefficient *η* which describes the rate of energy transfer between the adsorbate and the surface, with connection to the substrate heat bath being given by the stochastic term *ξ*(*t*)[144,192]. The former will describe single-particle motion, while for collective diffusion[188] the last term in Figure 10, which describes possible interaction forces **F**_*kj*_ with other adsorbates on the surface, ranging from simple site blocking to attractive and repulsive forces, needs to be considered.

A software package that provides a straight-forward way to expand the modelling of ultra-fast surface diffusion problems at the atomic scale based on solving the generlised Langevin equation has been made available by N. Avidor *et al.* under the GNU general public license[192].

### 3.4 Diffusion of water monomers on graphene

**Figure 11:**
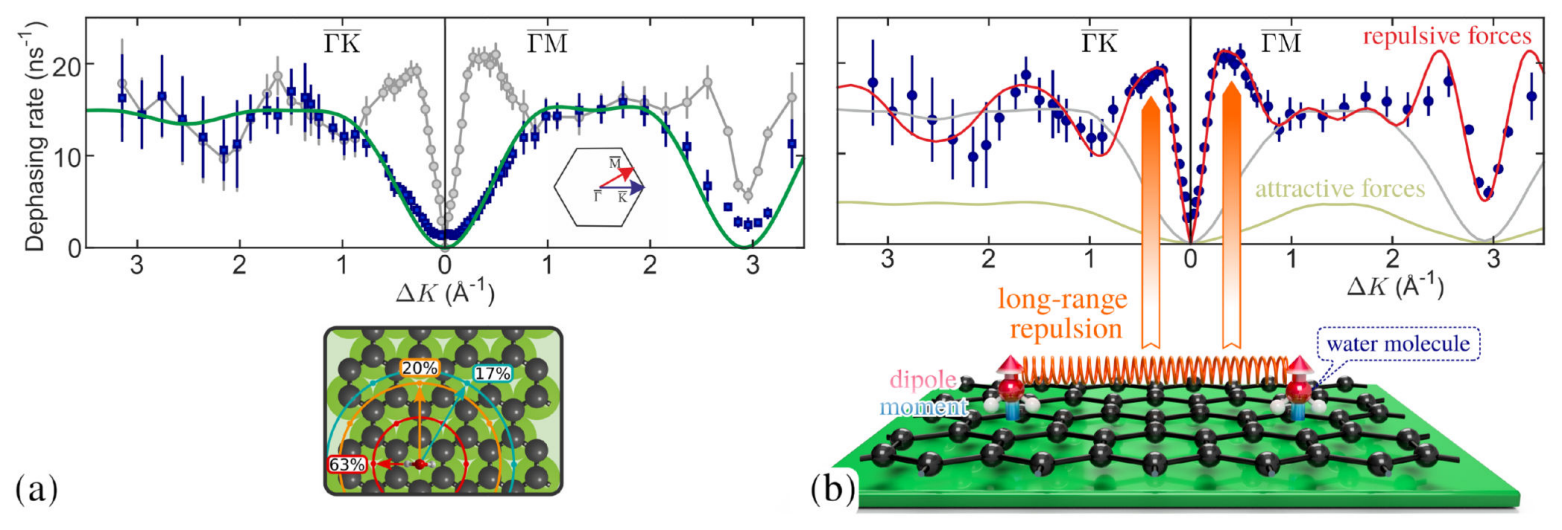
(a) HeSE measurements for the diffusion of water monomers on graphene. The momentum transfer dependence of the dephasing rate, *α*(Δ*K*), at 125 K from which the mechanism for diffusion follows. Blue data points show single-particle, or incoherent *α*(Δ*K*), deduced from the coherent scattering data ([56]). An analytical model (green curve, (3)) shows the expected behaviour for jumps between the centres of the graphene hexagons, as illustrated in the lower panel. (b) Comparison of the experimental dephasing rates for coherent scattering with kinetic Monte-Carlo (kMC) calculations (solid curves) provides conclusive proof for long-range repulsive interactions between the water monomers. Upon adding in the kMC a force to the hopping model derived in (a), the experimental data is described well by repulsive dipole forces (red curve) while models using attractive forces (green curve) or no forces (grey curve) cannot reproduce the data. Note that the model without forces (grey curve) is, as expected, similar to the analytic curve for incoherent scattering shown in (a). These forces can be attributed to dipolar interactions, arising from structural hindrance of water reorientation by the adsorption geometry – as illustrated by the blue/red arrow in the lower panel. The characteristic feature of these repulsive adsorbate interactions, as confirmed by kMC simulations, is a steep rise of the experimental data at 0.5 Å^−1^ as illustrated by the orange arrows. Reprinted from [56] under the terms of the Creative Commons CC BY license.

The only experimental study considering single molecular diffusion of water on graphene follows from HeSE measurements in reciprocal space, which allows to trace both tracer and collective diffusion as described in the following[56]. Within a small temperature window, individual water molecules diffuse in a dynamic equilibrium with islands of ice on graphene/Ni(111)[56] such that the molecules have life-times long enough to determine the scattering linewidth in the ISF *I* (Δ**K**, *t*). The grey points in Figure 11(a) show the variation of *α*(Δ*K*) for water motion at 125 K along both high-symmetry directions of graphene, at a relative coverage of 0.07 monolayers (ML)[56]. The Langevin description (3.3) provides the ability to distinguish between adsorbate-substrate and adsorbate-adsorbate interactions, i.e. between single particle and collective diffusion in the experiment[188]. The result for incoherent scattering and the corresponding single-particle dephasing-rate as shown by the blue points[56,188] is well described by the analytical CE-model (3), indicating that motion takes place by a jump mechanism over the periodic substrate: *α*(Δ*K*) is periodic in Δ*K* rising from the origin and returning to *α* = 0 at about Δ*K* = 2.9 Å^−1^ in the $\bar{\Gamma \text{M}}$ direction. The CE-model (3) (green curve) corresponds to jumps on the hexagonal graphene lattice, with the water molecule being adsorbed in the centre of the hexagon. Following a residence time of *τ* = (65 ± 3) ps, jumps occur to nearest, next-nearest, and second-nearest neighbours with relative jump contributions as illustrated in the lower panel of Figure 11(a).

According to (4), an accurate tracer diffusion coefficient, *D* = (4.1 ± 0.2) × 10^−10^ m^2^/s at 125 K is obtained, together with an activation energy, *E_a_* = (60 ± 4) meV from temperature dependent measurements using Arrhenius’ law (see Table 2). MD simulations of water clusters and nanodroplets obtained at much higher temperatures (room temperature)[143,193] are difficult to compare with the monomer rates and are indeed much higher. On the other hand, Ma *et al.* report *D* = 6 × 10^−9^ m^2^/s for MD simulations of water monomers at 100 K [62]. However, all calculations mentioned are performed on free standing graphene where the motion of ripples gives rise to ultra-fast diffusion[143] in contrast to the HeSE measurements on Gr/Ni(111) where those are suppressed[55].

Compared to the diffusion in (bulk) amorphous ice on the other hand, where for translational motion *D*_0_ is in the range of (0.5 − 5) 10^−17^ m^2^/s[194], or the diffusion of ASW at the liquid/ice interface with *D* ≈ 10^−21^ m^2^/s at 125 K[195], the diffusion of water monomers on graphene is incomparably faster. These results are in line with MD simulations by Ho and Striolo for the dynamical properties of water layers on graphene, reporting that in general, water surface diffusion is faster within the first monolayer than in the bulk[196].

We now turn to the interactions between water molecules, which are encoded in the differences between the coherent (blue data points) and incoherent rates (grey data points) in Figure 11(a). As reported by Tamtögl and co-authors these differences are caused by long-range repulsive forces between individual water molecules in the sub-monolayer regime, causing a kinetic energy barrier in the early stages of ice formation and clustering[56]. The behaviour is fundamentally different to ice formation at flat metal substrates, where attractive forces and hydrogen bonding have always been assumed. The nature of these intermolecular forces on graphene were determined from a series of kinetic Monte Carlo (kMC) simulations, with pairwise dipole-dipole forces between the H_2_O molecules[56], which are compared with the experimental data in Figure 11(b). The kMC simulations provide the ability to explore both repulsive and attractive interactions, with the red line in Figure 11(b) showing the case of repulsive interactions, where the dipole moment of each molecule had been adjusted to best fit the experimental data, giving a value of *p* = (1.8 ± 0.2) D[56].

As also shown by DFT calculations, and illustrated in Figure 11(b), individual water molecules adsorb with the same orientation, with a dipole moment slightly larger than for an isolated water molecule. The alignment of the dipoles perpendicular to the surface plane gives rise to strong repulsive interactions which manifests itself in the experimental *α*(Δ*K*) curve as shown schematically by the orange arrows.

Adsorbates repelling each other prefer a long-range quasi-hexagonal structure leading to a preferred, average distance between the adsorbates and reduced mobility on these length scales[50,144,197] while at the same time, when adsorbates approach each other their mobility increases compared to the non-repelling case. The result is a peak at lower Δ*K* (orange arrows) followed by a dip feature. While the grey line from the kMC simulations without interactions follows the same sinusoidal curve as the analytical expression, the case for inter-adsorbate repulsion (red line in Figure 11(b)) exhibits a peak appearing at low Δ*K* values due to the increased mobility at certain length scales, followed by a dip occurring at the length scale of the quasi-hexagonal arrangement.

These long-range dipole-dipole repulsion act to keep the water monomers apart representing a kinetic barrier to ice nucleation, at least in the low coverage regime and as long as no nucleation centres exist: In order for a water cluster to nucleate, molecules must first come into close proximity and must then re-orient to adopt a hydrogen bonded configuration. The reorientation barrier can be estimated at about 90 meV from DFT, while the mean kinetic energy of the substrate is only of the order of 10 meV. Here we note, that long-range repulsive interactions do not exclude the possibility of short-range attractive interactions which will instead occur within a length scale that corresponds to intra-cell diffusion. Scattering techniques can provide the full breadth of these microscopic details[198] while STM can only track the “visited” inter-cell diffusion sites.

Once the reorientation barrier is overcome, clustering and the onset of ice growth will commence with increasing coverage with hydrogen bonding becoming dominant. The latter may explain why attractive forces, have always been assumed and the significance of repulsion in the context of suppressing nucleation has not previously been recognised. While the adsorption geometry on graphene plays an important role, it seems reasonable to expect dipolar repulsion could occur more generally upon molecular adsorption[199]. Whenever dipoles are prevented from re-orienting, inter-adsorbate repulsion may suppress ice nucleation, suggesting anti-icing strategies[33,34,200] via enhancing the dipole formation e.g. using surface treatments leading to greater electron transfer.

### 3.5 Energy dissipation in monomer diffusion on TIs

**Figure 12:**
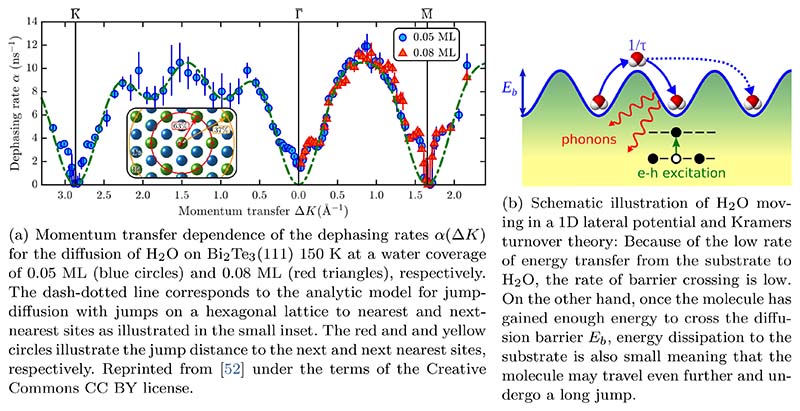
Molecular dynamics of water on novel surfaces: H_2_O on Bi_2_Te3.

In HeSE measurements of single water molecules diffusing on Bi_2_Te_3_(111), several similarities with the H_2_O/graphene measurements are found. Diffusion occurs via activated hopping motion (*E_a_* = 34 meV) on a hexagonal lattice with the jump distribution shown in Figure 12a and diffusion constants given in Table 2. There is again a signature for repulsive interactions between the individual water molecules, with the magnitude being however much smaller compared to graphene. Since the studied system provides a special platform for an atomic level investigation in what way energy is transferred between adsorbates and the substrate - because of the insulating interior the only contribution to electronic friction arises from the metallic surface state - we will instead concentrate our discussion on that topic.

At first glance the comparably low mobility of the water molecules seems to be in contrast with the significant number of long jumps (Figure 12a)[52]. In ideal Brownian diffusion, where there are no barriers, the rate decreases as the friction increases. However, if the diffusion is activated, as in the present case, then there exists also a low-friction regime where the rate decreases as the friction is reduced. Following from the rate of barrier crossing obtained in a Langevin description of dynamics in Energy dissipation, such a phenomenon is well understood in terms of Kramer’s turnover theory[201], as schematically illustrated in Figure12b. Because of the low rate of energy transfer from the substrate to the water molecule, the molecule obtains only seldomly enough energy to cross the diffusion barrier. On the other hand, once the molecule has gained enough energy to cross the barrier, energy transfer from the molecule back to the substrate is also small: It stays long enough in the “excited” state and there is a certain probability that it may travel even further and undergo a long jump.

Friction in surface diffusion processes can be caused by a variety of dissipative mechanisms, interactions with phonons and electrons in the substrate[202]. There is no simple way to disentangle the electronic from the phononic contribution to friction[202] but a low-friction scenario seems plausible for the current system: The phonon spectrum of Bi_2_Te_3_ suggests the absence of single-phonon coupling[52,203] while electron-hole excitations are restricted to the density of surface states arising from the topological character of the substrate[204] (see also Conclusion and open questions).

### 3.6 Experimental atomic-scale dynamics of other systems

#### Nanoscale mass transport of water monomers

We finish by comparing the activation energies and diffusion constants for single-molecule water diffusion on different substrates in Table 2 as reported at the date of writing this review. From the first reported measurements of monomer diffusion, *D* = 2.3 · 10^−23^ m^2^s^−1^ for diffusion at 40 K on Pd(111) was obtained[43], smaller than the later reported coefficient for Cu(111)[205]. The activation energies are all within the same order of magnitude except for *h*-TiO_2_(110), where the mobility of water monomers along the Ti troughs of the rutile *h*-TiO_2_(110) surface was determined[206]. Among all systems where the diffusion prefactor *D*_0_ are reported, water/Gr/Ni exhibits the highest *D*_0_, albeit experimental diffusion coefficients still tend to be slower than those extracted from MD simulations as mentioned above. Notably *E_a_* for graphene is almost twice as large as *E_a_* for Bi_2_Te_3_ with the latter having the larger attempt rate ϒ_0_, meaning that at low temperatures where mainly *E_a_* governs the diffusion rate H_2_O will move faster on Bi_2_Te_3_.

#### Further experimental reports

While we concentrate our review and comparison with experimental accounts to single-molecule water diffusion on other systems it should also be noted that dynamics of clusters etc. have been observed on flat metal substrates, including Pd(111)[43] and Cu(111)[205,207]. On Cu(111) trimer diffusion was observed at temperatures (< 10 K) where water monomers do not diffuse with the diffusion process following an inchworm-like manner [208]. In addition, diffusion rates for the self-diffusion on ice have been extracted from island formation mechanisms, giving an activation energy of 0.4 eV[209]. The latter should however not be compared to the monomer results reported in Table 2 and as already mentioned above, diffusion at the liquid/ice interface[195] tends to be much slower than monomer diffusion on graphene. Finally, apart from surface diffusion, it was shown that using STM confined water hexamers can be controllably switched[210], while quantum tunnelling of protons within water clusters can also be observed with STM/STS[46].

## 4 Conclusion and open questions

We reviewed recent progress in water adsorption and diffusion on surfaces, focusing on the behaviour of single water molecules at the sub-monolayer coverage on graphene, graphite and 2D materials, and limiting ourselves to a brief glace to the enormous amount of knowledge available on the formation of crystalline 2D ice on metal and non-metal surfaces. It is self-evident that the slow and difficult progress in determining the properties of water monomers on surfaces is due to two main factors: i) the strong hydrogen bonding between water molecules causes the rapid formation of water dimers and islands almost immediately upon water physisorbing on solid surfaces, therefore surface techniques generally employed to probe self-assembly on surfaces (STM and electron microscopies among others) are not suitable to investigate surface dynamics operating in the picosecond timescale; ii) water adsorbs on surfaces in a wide variety of conformations which easily interchange from one to another upon water diffusion forming hydrogen bonds. These conformations have only recently been accessible by theoretical investigations using DFT with semi-empirical or *ab-initio* dispersion corrections, long-range corrected functionals or range-separated exchange and correlation functionals.

The development of HeSE techniques has allowed for the first time to access with unprecedented detail the motion of single water molecules on perfectly flat crystal surfaces, including graphene and Bi_2_Te_3_. We have seen how the interplay between the dipole-dipole repulsion and the attractive dispersion and hydrogen bonding interactions creates a unique surface energy landscape which we have only just started to explore. Still, the more we learn about water, the more we find that unexpected complexities emerge.

Our understanding of water transport and assembly on the nanoscale is just starting to emerge: Fundamentally new aspects such as long-range repulisve forces and their significance in the context of suppressing nucleation provide implications for diverse fields, stimulating a wide range of new research, understanding and application. The atomistic origin of friction for water monomer diffusion and its role in determining the dynamics of water in contact with solid surfaces is also of paramount importance for technological applications, including water purification and desalination, separation, drug delivery and bio-detection. Here, much more detailed simulations and experiments will be required to resolve the importance of several effects towards energy dissipation: From the contribution of electronic and phononic friction to considering the full-dimensional potential of the diffusing molecule together with possible internal degrees of freedom.

As outlined, experiments providing insight into the atomic scale dynamics have so far solely been conducted on flat surfaces and at predominantly cryogenic temperatures. With respect to water and ice on cosmic dust grains, quantum tunnelling[211] and thus isotope effects might be an interesting perspective for conducting water dynamics measurements at low temperatures. Another rational step for future experiments is to go beyond flat interfaces to stepped surfaces and possibly consider the interaction of water with bio-structures adsorbed at surfaces. Yet it still seems to be quite a long way to molecular-level studies of “real” catalysts and in particular measurements at elevated temperatures which are relevant for catalysis and electrochemistry.

The continuous development in quantum chemical computational methods and, at the same time, the widespread availability and accessibility of high performance computing facilities has enabled the investigation of water at interfaces with fully atomistic quantum models. In the near future, we may expect that *ab-initio* molecular dynamics (AIMD) calculations of water monolayers and water nanodroplets will become feasible and replace current classical MD simulations. For sub-monolayer characterisation, more accurate methods than DFT have already started to be applicable to surface calculations and it might not be a far hope to be able to run Quantum Monte Carlo (QMC) calculations for water interfaces. The presence and role of quantum tunnelling in the self-assembly of water monolayers and in water monomers diffusion is still essentially unexplored and also in this case, the progress with approximated open quantum system treatments will allow us to go beyond simplistic tunnelling corrections[212].

Finally, it is worth noting that Machine Learning (ML) applications in quantum chemistry and surface physics have made huge advancement in the last decade with progress in the ability of producing highly accurate potential energy surfaces[213], transition states[214] and predicting atomic-scale surface properties [215]. To say that ML could transform our ability to do science and in particular the way we do quantum chemical calculations is indeed an understatement, and we can see that in the realm of water surface dynamics the ML revolution has already, slowly, starting to produce results.

## Figures and Tables

**Table 1:** Summary of DFT calculations for a single H_2_O molecule on a graphene surface, with adsorption energy (*E_ads_*) and adsorption distance *d*. Preferential adsorption site are hollow (H) and top (T) with the orientation being with both H atoms pointing downwards (d) or one H atom parallel to the surface (v).

Site	Orient.	*E_ads_* (meV)	*d* (Å)	Ref.
H	v	47	3.5	[69]
H	d	98	3.42	[62]
H	d	99	3.37	[66]
H	d	116	3.28	[70]
H	d	124	3.36	[71]
T	d	135	3.23	[72]
H	d	138	3.26	[73]
H	d	161	3.2	[67]
H	d	123	2.55	[74]^1^
H	d	183	3.21	[75]^1^

1 Values for graphene/Ni(111)

**Table 2:** Comparison of experimentally determined diffusion parameters for water monomers on different substrates, including the activation energy, *E_a_*, the diffusion constant, *D*_0_, in Arrhenius pre-exponential form, and the hopping attempt rate, ϒ_0_.

Substrate	Adsorbate	*E_a_* (meV)	*D*_0_ (m^2^s^−1^)	ϒ_0_ (s^−1^)	*T* range (K)	Ref.
Graphene/Ni(111)	H_2_O	60	1.1 · 10^−7^	4.0 · 10^12^	113 – 130	[56]
Cu(111)	D_2_O	75	1.8 · 10^−8^	1.8 · 10^11^	23 – 29	[205]
Bi_2_Te_3_(111)	H_2_O	34	1.3 · 10^−8^	1.7 · 10^11^	130 – 160	[52]
NaCl(001)/Ag(111)	D_2_O	149	1.5 · 10^−8^	1.0 · 10^12^	42 – 52	[17]
Pd(111)	H_2_O	126	–	1.0 · 10^12^	40 – 55	[43]
*h*-TiO_2_(110)	H_2_O	460	–	4.0 · 10^10^	170 – 210	[206]

## Footnotes

1 Eiseley L. The Immense Journey, Random House, New York, 1957. Chapter: The Flow of the River, p 15
